# Forecasting Natural Gas Prices Using Wavelets, Time Series, and Artificial Neural Networks

**DOI:** 10.1371/journal.pone.0142064

**Published:** 2015-11-05

**Authors:** Junghwan Jin, Jinsoo Kim

**Affiliations:** Department of Natural Resources and Environmental Engineering, Hanyang University, Seoul, Korea; Universidad Veracruzana, MEXICO

## Abstract

Following the unconventional gas revolution, the forecasting of natural gas prices has become increasingly important because the association of these prices with those of crude oil has weakened. With this as motivation, we propose some modified hybrid models in which various combinations of the wavelet approximation, detail components, autoregressive integrated moving average, generalized autoregressive conditional heteroskedasticity, and artificial neural network models are employed to predict natural gas prices. We also emphasize the boundary problem in wavelet decomposition, and compare results that consider the boundary problem case with those that do not. The empirical results show that our suggested approach can handle the boundary problem, such that it facilitates the extraction of the appropriate forecasting results. The performance of the wavelet-hybrid approach was superior in all cases, whereas the application of detail components in the forecasting was only able to yield a small improvement in forecasting performance. Therefore, forecasting with only an approximation component would be acceptable, in consideration of forecasting efficiency.

## Introduction

The development of unconventional gas in the United States accelerated through the 2000s, and contributed up to $284 billion to the US GDP in 2012. Moreover, millions of jobs in the US are supported by the unconventional oil and gas value chain [1]. These changes in the US introduced structural changes to the energy market, such as the decoupling of oil and natural gas prices. Therefore, the attention given to natural gas has been increasing. Furthermore, since the accident at the Fukushima nuclear power plant, increasing the supply of natural gas has become more important, especially in East Asia. This can be seen from the sharp rise in the spot Liquefied Natural Gas (LNG) demand and increased average distance of LNG transportation in Asia [2]. Based on this background, the International Energy Agency raised the prospect of a golden age of natural gas, in which the supply of gas will exceed the supply of oil and coal [3, 4].

The natural gas market can be separated into two broad groups: The first comprises the markets of North America and the United Kingdom, and the other represents Europe and Asia. The market price in North America and the United Kingdom is dictated by supply and demand conditions, whereas that of Asia and Europe is indexed to the oil price [5]. Consequently, many studies of oil prices have been conducted [6–8], but there are relatively few studies on natural gas prices. However, the question on decoupling arose, because coupling trends differ between markets [9, 10]. In addition, the trade of natural gas in the past involved mainly long-term contracts, whereas short-term contracts and spot trading have recently increased [11]. Therefore, accurate natural gas price forecasting is becoming more important for Asian countries.

In general, gas prices are much higher in Asia than in other regions, due to higher storage prices, delivery distances, and immature market conditions. Therefore, developing a competitive natural gas market for spot and futures, and a trading hub in East Asia, is proposed as a method for solving the problem of high prices [12]. To improve the efficiency of the newly introduced market, short-term forecasting is necessary for natural gas spot prices. In addition, accurate natural gas predictions are desirable to support effective investment decisions, negotiate import and export contracts, and draft energy mix policies. In this regard, we perform a short-run price forecast for natural gas using a wavelet decomposition method.

The most common models in time series forecasting, particularly in the parametric estimation method, are the autoregressive integrated moving average (ARIMA) and generalized autoregressive conditional heteroskedasticity (GARCH) models. ARIMA is a basic linear forecasting model, which uses a lagged series. Because of its simplicity and good performance, ARIMA has been applied to many time series analyses [13–16]. GARCH is based on the idea of non-consistent variance in a general time series, and can be applied to the volatility analysis of a time series [17–19]. Recently, machine-learning methodologies, such as artificial neural networks (ANN), have been employed in many forecasting studies [20–22], as the versatility of these models allows them to be applied to any time series data. Because ANN is not based on an asymptotic theory in econometrics, it has a wide range of applications, and as such, its use is increasing.

Since the 2000s, wavelet decomposition has been combined with time series models as a preprocessing method. Wavelet decomposition (or wavelet transform) decomposes time series data into approximation and detail components, so that different forecasting models can be applied to each component. This property can improve the performance of forecasting. The validity of this approach has been proved in various studies. Yousefi *et al*. [23] used wavelet decomposition in a numerical analysis to test the efficiency of futures markets. New York Mercantile Exchange (NYMEX) futures from one month to four months were compared to West Texas Intermediate (WTI) spot market prices, forecasted by models using wavelet decomposition and numerical analysis models. The comparison of the resulting correlation coefficients showed a higher correlation in the forecasted values than in futures prices. Tan *et al*. [24] constructed a forecasting model using wavelet decomposition and ARIMA and GARCH models to forecast the electricity price, using the market clearing price and the locational marginal price of Spain’s electricity market. The results showed an improvement in the forecasting outcome over the findings of other studies. Moazzami *et al*. [25] applied wavelet decomposition and ANN to seasonal data for Iran’s national grid, to forecast the day-ahead peak load. Three types of ANN algorithms were used. A model that combined a generalized feed forward neural network with wavelet decomposition yielded the best results. Mellit *et al*. [26] used wavelet decomposition and ANN to forecast the total solar radiation from 1981 to 2001. A comparison of the results demonstrated that the suggested model was superior for the forecasting of total solar radiation to autoregressive, ARIMA, the Markov transition matrix, and the multi-layer perceptron network. Liu *et al*. [27] used wavelet decomposition, wavelet packet decomposition, and ANN to forecast wind speed. ARIMA, ARIMA-ANN, and Neuro-Fuzzy models were compared with the suggested models, and the wavelet packet Broyden-Fletcher-Goldfarb-Shanno provided the best forecasting results. Soltani [28] combined wavelet decomposition with ANN to forecast Mackey–Glass time series and sunspot data. The forecasting results showed an improved performance, compared to models used in other studies. Ahmad [29] used fuzzy wavelet decomposition to predict IBM daily prices, NASDAQ daily index values, and S&P 500 daily index values. Wavelet decomposition showed a better forecasting performance when the noise was removed, i.e., when denoising was applied. Shafie [30] applied wavelet transform, ARIMA, and Radial Basis Function Neural Networks (RBFN) to forecast the price of electricity in Spain by treating the price behavior as a nonlinear function, which therefore required a nonlinear model to capture the behavior of the price. He decomposed data by wavelet decomposition and four decomposed series were recognized by ARIMA. After applying ARIMA, inverse wavelet transform was performed and then the RBFN network was used to correct the errors of the wavelet-ARIMA predictor. RBFN detected potential nonlinear patterns hidden in the residual term. From the comparison with some of the most recent price forecasting methods, the proposed method showed a considerable improvement in the forecasting accuracy. Pindoriya [31] used an adaptive wavelet neural network (AWNN) for short-term price forecasting in the electricity markets. WNN was first proposed as an alternative to the classical feed-forward neural network (FFNN) for approximating arbitrary nonlinear functions. In addition, the Mexican hat wavelet is used as the mother wavelet, as opposed to the Daubechies wavelet, which is the most popular approach. This is because the Mexican hat wavelet is more suitable for continuous wavelet transform, which is needed for adaptive WNN. In this study, the forecasting results of the locational marginal price and market-clearing price based on the AWNN model were more accurate than the forecasting results obtained by other models.

From these studies in the literature, we could expect that any data sets, such as the oil price and stock prices, may be suitable for wavelet decomposition. The properties of wavelet decomposition, which extracts low and high frequency components from the original data, allow each component to be analyzed more easily. Furthermore, ARIMA, GARCH, and wavelet decomposition are linear models and ANN is a nonlinear model. In other words, wavelet decomposition can handle a noise and/or shocks and ARIMA, GARCH, and ANN can capture various movements in time series. Therefore, we can expect that the difference models will play a complementary role to describe data [32, 33] and the forecasting power will increase consequently when using the combination of these methods. The properties of wavelet decomposition are discussed in more detail in Section 3 (methodology).

When we forecast a time series, future information should not be implied based on past time points. However, some wavelet transformations, because of their own structures, such as Daubechies and Symlets, use future information in addition to previous information. This property of wavelet transformations is not appropriate for forecasting subsequent periods. To the best of our knowledge, the only wavelet decomposition method that has no structural problem when transferring future information to the present is the Harr wavelet. We refer to this as the boundary problem. There have been some studies of this problem, and they have used two approaches to solve it [34, 35]; the use of the Harr wavelet is one such approach. Owing to the shape of the Harr wavelet, only previous and present time point data are used. The other approach is to use a time-based wavelet transform, which incrementally transforms data from the starting point to the *n*th point. In this study, we applied a modified version of this time-based wavelet transform, because the Daubechies wavelet is more appropriate to our time series. We avoided the boundary problem by adjusting the decomposition period.

Based on this body of literature, our impression was that combining wavelet decomposition with other forecasting models as a preprocessing step would improve the forecasting results. Actually, in all of the above studies in the literature, the researchers compared the results they obtained with their hybrid model with those of traditional models and in all cases, it was shown that the combined wavelet model produced superior results. This approach is quite similar to a denoising process. As mentioned above, wavelet decomposition separates data into several series. In this process, some of the components that complicate the use of the model are eliminated, which facilitates data analysis by forecasting models such as ARIMA, GARCH, and ANN. Other researchers’ promising results [25–27, 36] demonstrating the advantages of wavelet decomposition prompted us to exploit the advantages of wavelet decomposition as a preprocessing process.

Furthermore, Ahmad [15] focused on wavelet decomposition in his study of the denoising process. In his study, the time series was decomposed into approximate and detail components by implementing wavelet decomposition. The approximate component pertains to a trend of the time series, and the details represent a kind of variance. If the time frequency is very short, these details can then be regarded as noise. Consequently, excluding detail components from the forecasting analysis can improve the performance, provided that the detail components simply represent noise.

We test this hypothesis by comparing forecasting results including detail components with those that do not. Among the above-mentioned studies, there is no analysis related to natural gas spot prices, or the short-term behavior of natural gas prices. Because of the increasing importance of natural gas as an energy source, accurate forecasting of the natural gas spot price is necessary, as discussed above. In addition, we find that there are ambiguous applications of wavelet decomposition in comparisons of multi-step forecasting results. Therefore, we forecast the natural gas spot price, and discuss the boundary problem when applying wavelet decomposition. The Henry Hub weekly spot price covering the period from January 2000 to November 2013, obtained from the Energy Information Administration (EIA), is used. The data are available on http://www.eia.gov/dnav/ng/hist/rngwhhdW.htm. The forecasting performances of ANN, ARIMA, and of each model combined with wavelet decomposition are compared from one-step ahead to four-step ahead. These methods are implemented twice: first without detail components, and then with detail components, which is modeled by GARCH. Furthermore, these analyses are performed in two different ways, a case where wavelet decomposition is correctly applied, and one in which the boundary problem is ignored. We show that, if the boundary problem is ignored, the forecasting results could yield an overestimate. Therefore, we can clearly identify the effect of detail components and the boundary problem.

The remainder of this paper is organized as follows. Section 2 details the overall framework of this study. In Section 3, we state the methods used in this study, and the boundary problem. Section 4 presents the forecasting results for two cases: the first in which the boundary problem is considered and in the other in which it is disregarded. In Section 5, we present a summary of our results and conclude the paper.

## Research Framework

**Fig 1:**
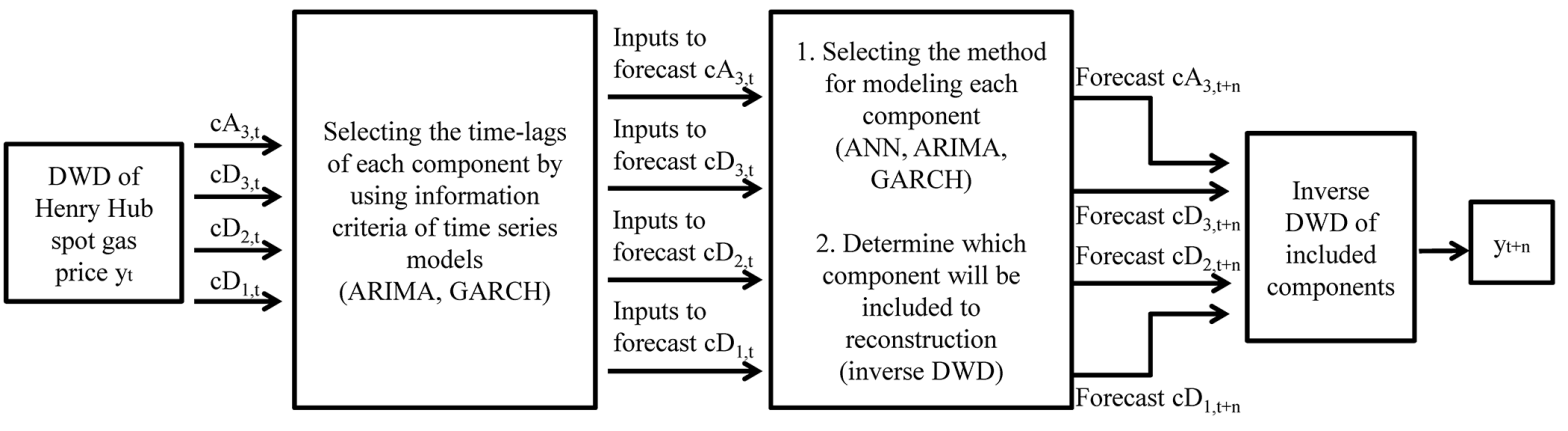
Flow chart of the study.

This paper employs wavelet decomposition and the ARIMA, GARCH, and ANN models, to forecast the Henry Hub weekly gas spot price. The importance of the boundary problem of wavelet decomposition is illuminated by applying two different decomposition approaches. In addition, the effect of detail components in forecasting is analyzed by comparing the forecasting results with or without the detail components. ARIMA and ANN are used to forecast approximation components, while ANN and GARCH are used to forecast detail components. A flowchart of this study is presented in Fig 1, and we designed our experiment in two ways to compare the boundary problem issue. The experiment 1 doesn’t consider the boundary problem, whereas the experiment 2 does. The detailed framework is described as follows:

### Experiment #1

1. Use discrete wavelet decomposition (DWD) to decompose the Henry Hub daily gas spot price into a number of approximations and detail component series. We carry out a level 3 DWD, in which we use one approximation component and three detail components. Approximation components can be considered as the main movements or trends of the original series. Detail components might represent volatility or noise of the original series. For the comparison, we apply the DWD in two ways: modifying the decomposition period to correct the boundary problem, and implementing the decomposition as is.
2. Group the decomposed series into a training period and a testing period. After that, determine the lags in the approximation and detail components by using the Akaike Information Criterion for the ARIMA and GARCH models, respectively. The number of hidden nodes of ANN applicable to each of the decomposed subseries is determined by one-step ahead forecasting Mean Squared Error (MSE).
3. Apply the forecasting methods for each component. In this step, we can show the overestimated results caused by ignoring the boundary problem. The basic ARIMA and ANN models are applied in this step, because the main aim of this step is to reveal the overestimated results caused by the boundary problem.
  - Use ANN with DWD to forecast approximation without including detail components. This means that we assume detail components are noise.
  - Use ARIMA with DWD to forecast approximation without including detail components, which is the same as case A.
  - Apply ANN to the approximation and details for forecasting. In this case, we include detail components to obtain the results, and this means that these components are necessary factors to capture the volatility of the original series. The numbers of detail components that are included in the forecasting models vary from one to three. In other words, we set up three different forecasting models: one model with only one detail component, another model with two, and a third model with all three of the detail components.
  - The above three cases are calculated with the aim of comparing the forecasting results which were obtained by adjusting the wavelet decomposition of the next step. In other words, the above three cases are performed to demonstrate the effect of overestimating. The other purpose of our study, which is to show the effects of the inclusion of detail components, was achieved by applying ARIMA and ANN to forecast the approximation component, and ANN and GARCH for the detail components. In this step, we assume that the detail components represent a random walk process, with heteroskedasticity and mean zero. This assumption is based on the results of Engle’s test [37] and Reboredo and Rivera-Castro [38] and it is quite natural to assume that way when we see the volatility of detail components. In this case, we did not use ARIMA to forecast details. Because GARCH and ANN are more suitable to model the volatility series than ARIMA, we thought there is no need for using ARIMA to forecast volatility. The results obtained in this stage are expected to enable the effects of detail components to be identified.
4. Reconstruct the forecasted series, and compare the results. Subsequently, expect to deduce whether the detail components for the Henry Hub natural gas spot price represent dispensable noise, or are a necessary ingredient for improving the forecasting performance.

### Experiment #2

1. Adjust the method of applying wavelet decomposition to correct the boundary problem, and repeat steps (3) and (4) in the experiment #1. Comparing the results in this step is expected to reconfirm the best combination of wavelet components and forecasting models.

## Decomposition and Forecasting Methods

### Discrete wavelet decomposition

DWD is a preprocessing method that projects a time series onto a collection of orthonormal basis functions. This transformation is applied to the data to obtain further information from the time-domain original data. After applying DWD to the data, we can analyze signals by decomposing them into various frequencies. The high-frequency component may be noisy, but the low-frequency component would contain a clear pattern of the original data, which facilitates forecasting. In this study, DWD is used to decompose the Henry Hub weekly spot prices into four subseries. DWDs consist of two basic wavelet functions, the father wavelet *φ*, and mother wavelet *ψ* [39, 40]:

$$\varphi_{\text{j},\text{k}}\;(\text{x})=2^{-\text{j}/2}\;\varphi (2^{j}t-k)$$ (1)

$$\psi_{\text{j},\text{k}}\;(\text{x})=2^{-\text{j}/2}\;\psi (2^{j}t-k)$$ (2)

The parameter *j* = 1,…,*J* is the scaling parameter in the *J* ^th^ level of decomposition, and *k* is a translation parameter.

The father wavelet transforms the original signal (*y*(*x*)) to an approximation component *D*, and the mother wavelet transforms it to the detail component *A*, which is similar to the smoothed original data [41, 42]. Details are associated with oscillations of lengths 2–4, 4–8,…, 2^*j*^-2^*j+1*^. We write the transforms as follows:

$$D_{\text{j},\text{t}}=\int_{-\infty }^{\infty }\text{y}(\text{x})\varphi_{\text{j},\text{t}}\;(\text{x})\text{dx}$$ (3)

$$A_{\text{j},\text{t}}=\int_{-\infty }^{\infty }\text{y}(\text{x})\psi_{\text{j},\text{t}}\;(\text{x})\text{dx}$$ (4)

**Fig 2:**
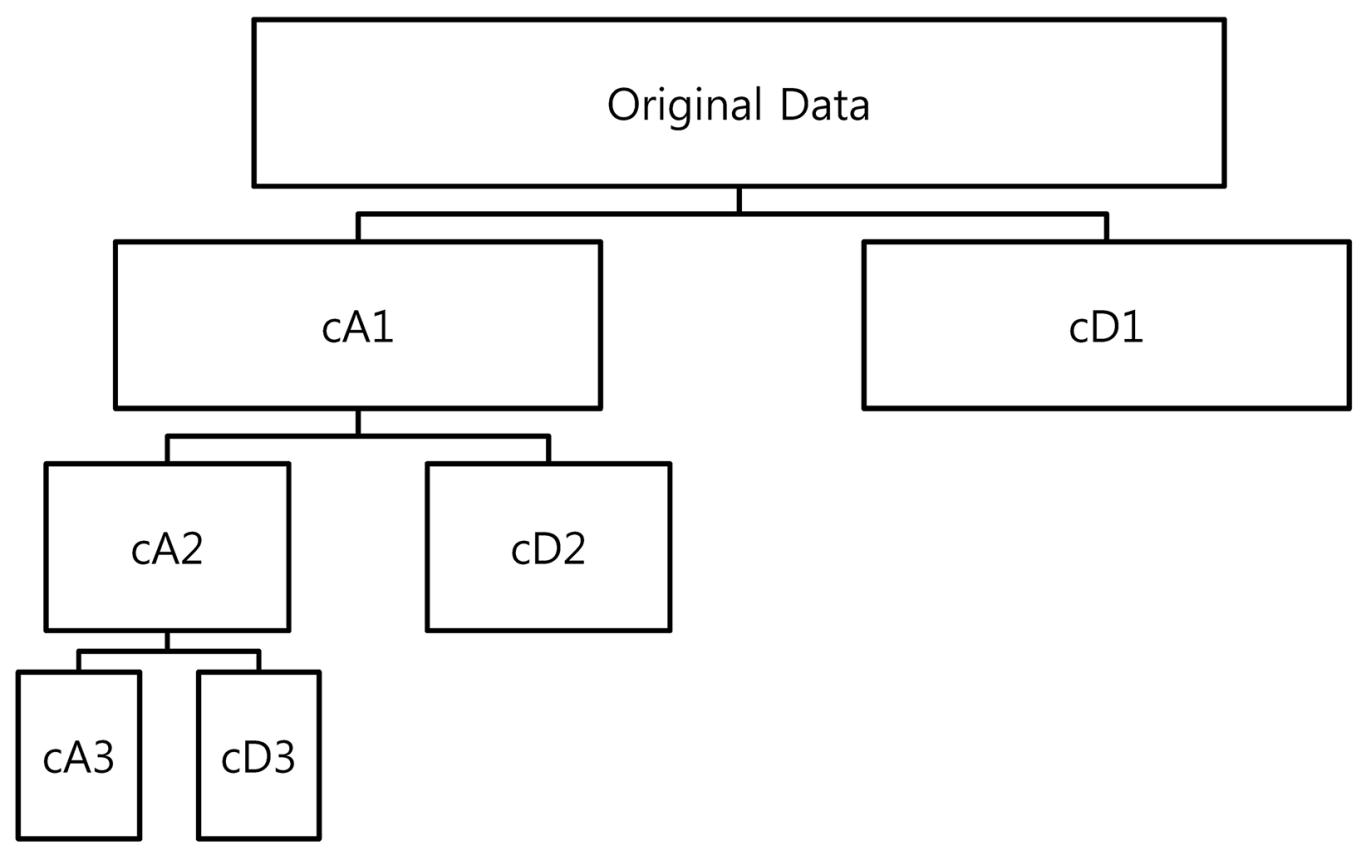
Structure of wavelet decomposition.

The structure of wavelet decomposition is presented in Fig 2, where *j* is the decomposition level. From this structure, we infer that the original signal is represented by the summation of the decomposed components. Therefore, we can represent the original signal as the combination of approximation and detail components, as follows:

$$y(x)=A_{j}(x)+D_{j}(x)+D_{j-1}(x)+\ldots +D_{1}(x)$$ (5)

After the decomposition, we can apply various forecasting methods for each component to draw the best forecasting performance.

### Decomposition results

**Fig 3:**
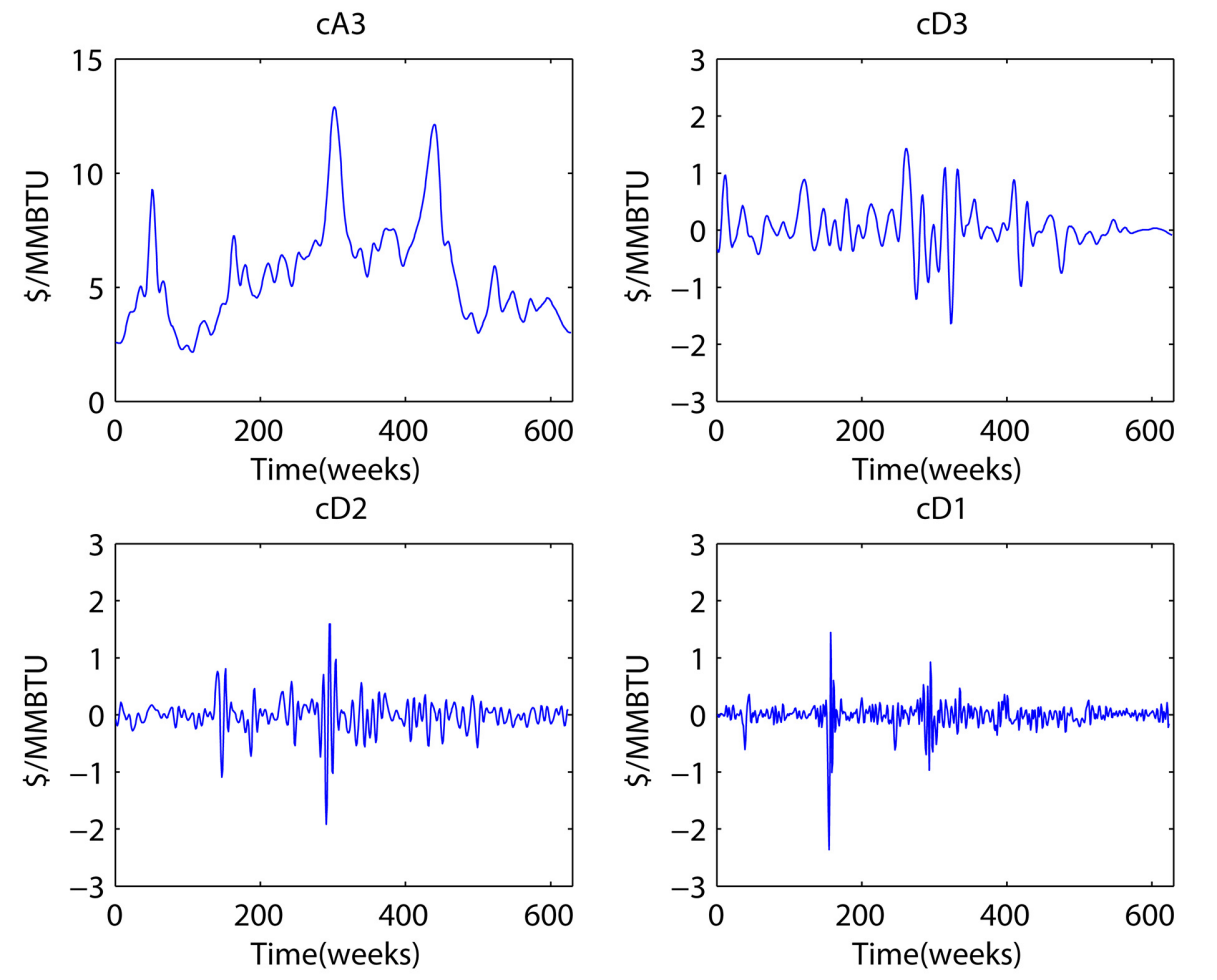
Decomposed components.

Fig 3 shows the components generated by discrete wavelet decomposition. Clockwise from the upper left, we see the approximation component decomposed three times, whereas the detail component is shown decomposed three times, one time, and two times, respectively. This discrete wavelet decomposition can be carried out by the Daubechies, Coiflets, Symlets, or Discrete Meyer approach, among others. Among these wavelets, Daubechies and Symlets can be used for a perfect reconstruction with the maximum number of vanishing moments. Symlets are perfectly symmetrical; Daubechies are not. Because symmetry could decrease the flexibility in expressing data, we choose Daubechies wavelets. The number of vanishing moments of Daubechies wavelets was determined as three for the best MSE, within range of perfect reconstruction available.

### ARIMA and GARCH

We used the traditional time-series models, ARIMA and GARCH, as control groups to compare the performance of the forecasting results. ARIMA is the most general linear model and consists of auto-regressive terms AR(p) and moving-average terms MA(q).

$$Y_{t}=\varphi_{1}Y_{t-1}+\varphi_{2}Y_{t-2}+\varphi_{3}Y_{t-3}+\ldots +\varphi_{p}Y_{t-p}+\delta +\varepsilon_{t}-\theta_{t-1}\varepsilon_{t-1}-\theta_{t-2}\varepsilon_{t-2}-\ldots -\theta_{t-q}\varepsilon_{t-q}$$ (6)

If there is a need for the difference to be rendered stationary, i.e., stationarized, the difference term should be included. For Eq 6, we can use back shift operator *B* instead of lagged variables.

$$\varphi_{p}(B)\nabla^{d}Y_{t}=\delta +\theta_{q}(B)\varepsilon_{t}$$ (7)

∇^*d*^ can also be rewrote using back shift operator and then we can get the following general ARIMA equation, Eq 8. Eq 8 is the general expression of ARIMA(p,d,q), where *Y* is the original time series, *B* is the back shift operator, and *d* is the number of differences [43, 44].

$$\varphi_{p}(B){\left(1-B\right)}^{d}\;Y_{t}=\delta +\theta_{q}(B)\varepsilon_{t}$$ (8)

Data with heteroscedasticity could be described by ARCH(q) model suggested by Engle. The following Eq 9 is a general representation of ARCH(q) model.

$$\sigma_{t}{}^{2}=\alpha_{0}+\alpha_{1}\varepsilon_{t-1}^{2}+\alpha_{2}\varepsilon_{t-2}^{2}+\ldots +\alpha_{q}\varepsilon_{t-q}^{2}$$ (9)

GARCH is a generalized version of ARCH like ARIMA which is generalized form of ARMA. GARCH is useful model to analyze the volatility of a signal. This model is similar to ARIMA and comprises variance and white noise. The following equation is the general expression of GARCH(p,q) [43, 44].

$$\sigma_{t}{}^{2}=\alpha_{0}+\delta_{1}\sigma_{t-1}^{2}+\delta_{2}\sigma_{t-2}^{2}+\ldots +\delta_{q}\sigma_{t-q}^{2}+\alpha_{1}\varepsilon_{t-1}^{2}+\alpha_{2}\varepsilon_{t-2}^{2}+\ldots +\alpha_{q}\varepsilon_{t-q}^{2}$$ (10)

### Artificial Neural Network (ANN)

**Fig 4:**
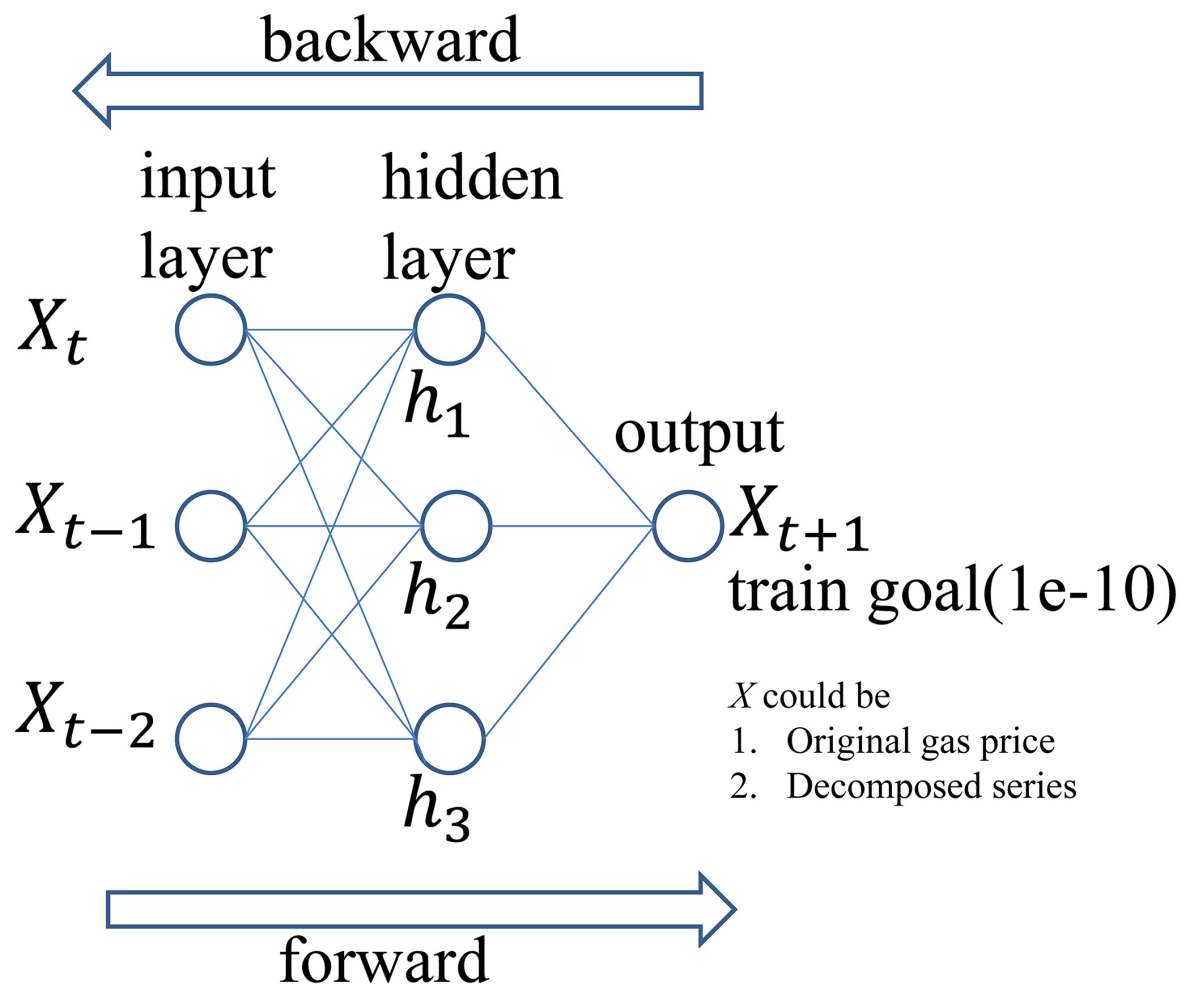
Flowchart of multilayered perceptron back-propagation neural networks.

ANN is a mathematical model that imitates the human brain to solve problems. Because ANN has the ability to train itself under various circumstances, various fields such as finance and marketing make use of this method [36, 45–47]. In this study, multilayered perceptrons (MLP) are used as a forecasting model, and back propagation is used as a training algorithm. MLP is a layered feed forward network that is trained by static back propagation. Each perceptron has several inputs, and one output that is a nonlinear function of the inputs. Back propagation algorithms are most frequently used in the MLP model. Back propagation modifies the connection strength between the output nodes and the inner nodes. There are several advantages to this method. It is easy to use, and it can model any type of data. Its disadvantages are that the time taken for training is longer than that of other methods, and that it requires large amounts of training data. In short, networks with one hidden layer are capable of approximating any continuous functional mapping, if the number of hidden units is sufficiently large. The flow of the algorithm used in this paper is presented in Fig 4. The numbers of inputs, outputs, and hidden nodes are selected empirically, or by the Akaike informative criterion.

### ANN model specification

**Fig 5:**
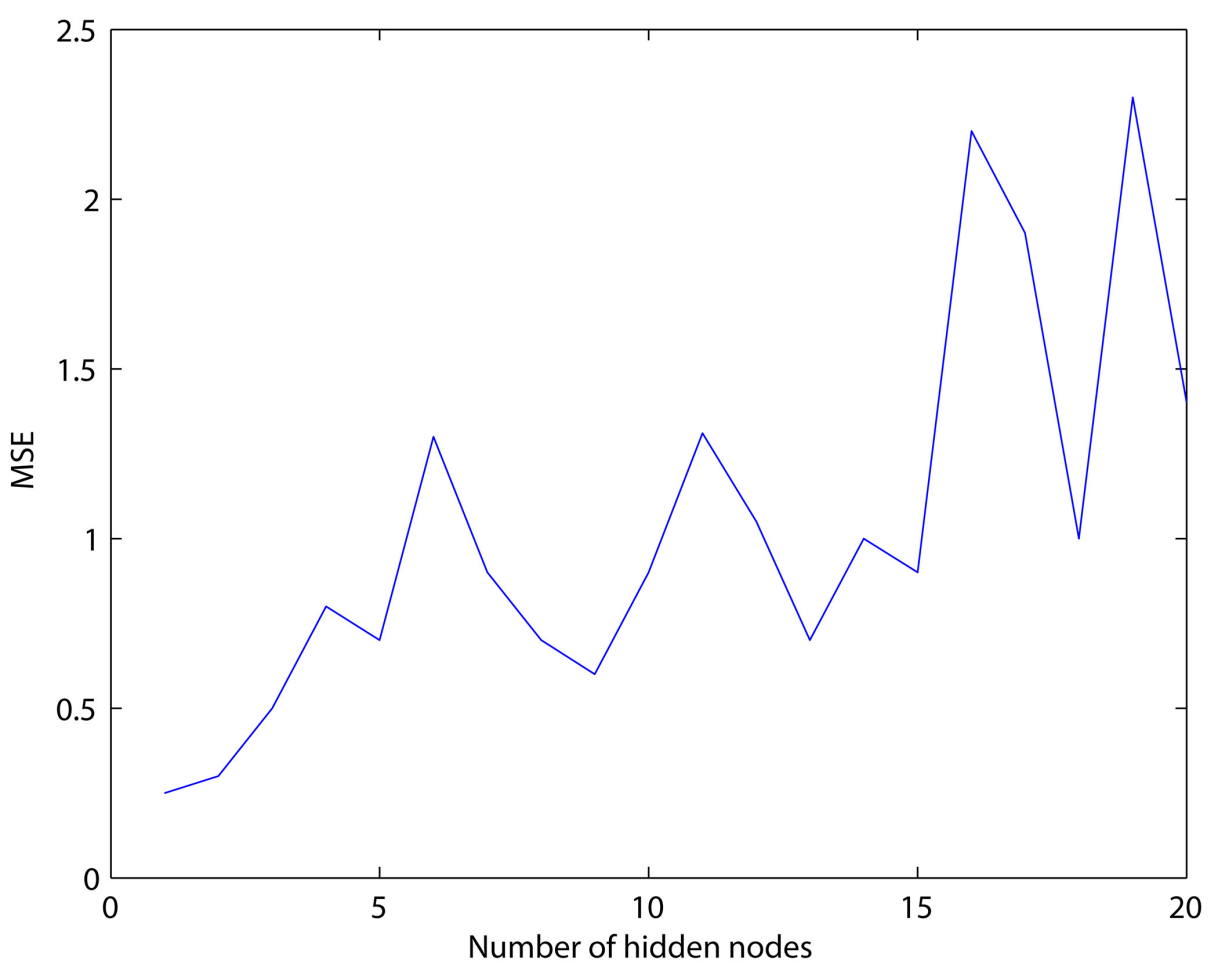
MSEs with the number of hidden nodes.

We used the Levenberg-Marquardt back propagation method for our ANN model. For this ANN model specification, we find that the most influential element in ANN forecasting is the number of hidden nodes. The number of input factors is selected based on the information criterion (AIC) of the time series models. The number of lags for the original data and the cA3 component are determined by AIC for ARIMA specification while the number of lags for the cD1, cD2, and cD3 components is determined by AIC for GARCH specification. According to Reboredo and Rivera-Castro [38], the detail component represents the volatility of a time series. Shumway and Stoffer, and Francq and Zakoian [43, 48], suggest that the GARCH model is suitable for modeling this volatility. Therefore, the lags of the detail component are selected based on the GARCH model. The number of hidden nodes for both the original and decomposed series is selected by MSE, which is calculated from one-step ahead forecasting of the training set, because of its general use for checking the performance of the model [49]. Fig 5 demonstrates the MSE behavior when the number of hidden nodes changes in the original gas price series As seen in Fig 5, the use of one and two hidden node was found to be the best and the second best. However, these nodes did not satisfy the condition of training which means model was not converged. We repeatedly ran our model varying the epochs and performance goal but those models could not be converged even in the condition of over three thousand epochs and 10^−9^ performance goal. On the other hand, the model with three nodes was converged at one thousand epochs and 10^−10^ performance goal. Since an excessive number of training epochs could lead inappropriate estimation [50] and our goal is to validate the usefulness of the wavelet decomposition for the linear (ARIMA, GARCH) and non-linear (ANN) forecasting models, we selected the three nodes model as the optimal ANN model. In this way, the optimal number of hidden nodes for each component was determined. The optimal numbers of hidden nodes for the other components are listed in the Table A in S1 File.

### Adjusted time-based wavelet decomposition

If one does not take the boundary problem into account when conducting wavelet decomposition analysis, an overestimation problem is allowed to arise; hence, the results will be not credulous. As stated in the previous section, the boundary problem in applying wavelet decomposition to a time series is an important issue. Nguyen and Nabney [34] approached this problem, and introduced appropriate wavelets in order to solve it. Certain wavelets use future information about a time series in the decomposition, and this is not reasonable in the concept of forecasting. They solved this problem by using Harr wavelets, which only use information from past and present data. Shensa [35] also solved this problem, by using an à trous time-based wavelet decomposition. We consider the Harr wavelet to be somewhat inflexible in comparison to other wavelets. Because we want to express the data in a flexible manner, an à trous time-based decomposition seems to be more suitable, in spite of the fact that it is a more time-consuming process. The adjusted time-based wavelet is based on à trous time-based decomposition. This is a simpler and less time-consuming process, but only even step decomposition is available, due to the properties of discrete wavelet decomposition. The procedure we applied is as follows.

Consider a series S(1), S(2),…, S(100) for two-step forecasting, and perform the following steps:

1. Carry out the discrete wavelet decomposition on S(1), S(2),…, S(k).
2. Retain the decomposed values for the *k-1* ^th^ and *k* ^th^ time points only.
3. If k < 100, set k = k + 2 and repeat these steps.

Following this procedure, we could reduce the process to half of the à trous time-based decomposition.

## Forecasting Results

As mentioned in the section on the research framework, we applied ARIMA and ANN to the approximation component, and ANN and GARCH to the detail components, for forecasting. When the detail components were included in the forecasting framework, we varied the number of detail components in the model. This is because we wanted to determine which of the detail components are relevant for forecasting, and which could be assumed to be noise. Although the approximation component is the most important for expressing the original data, if the detail components are not noise, then including those components in the model could improve the forecasting performance.

### Data

**Fig 6:**
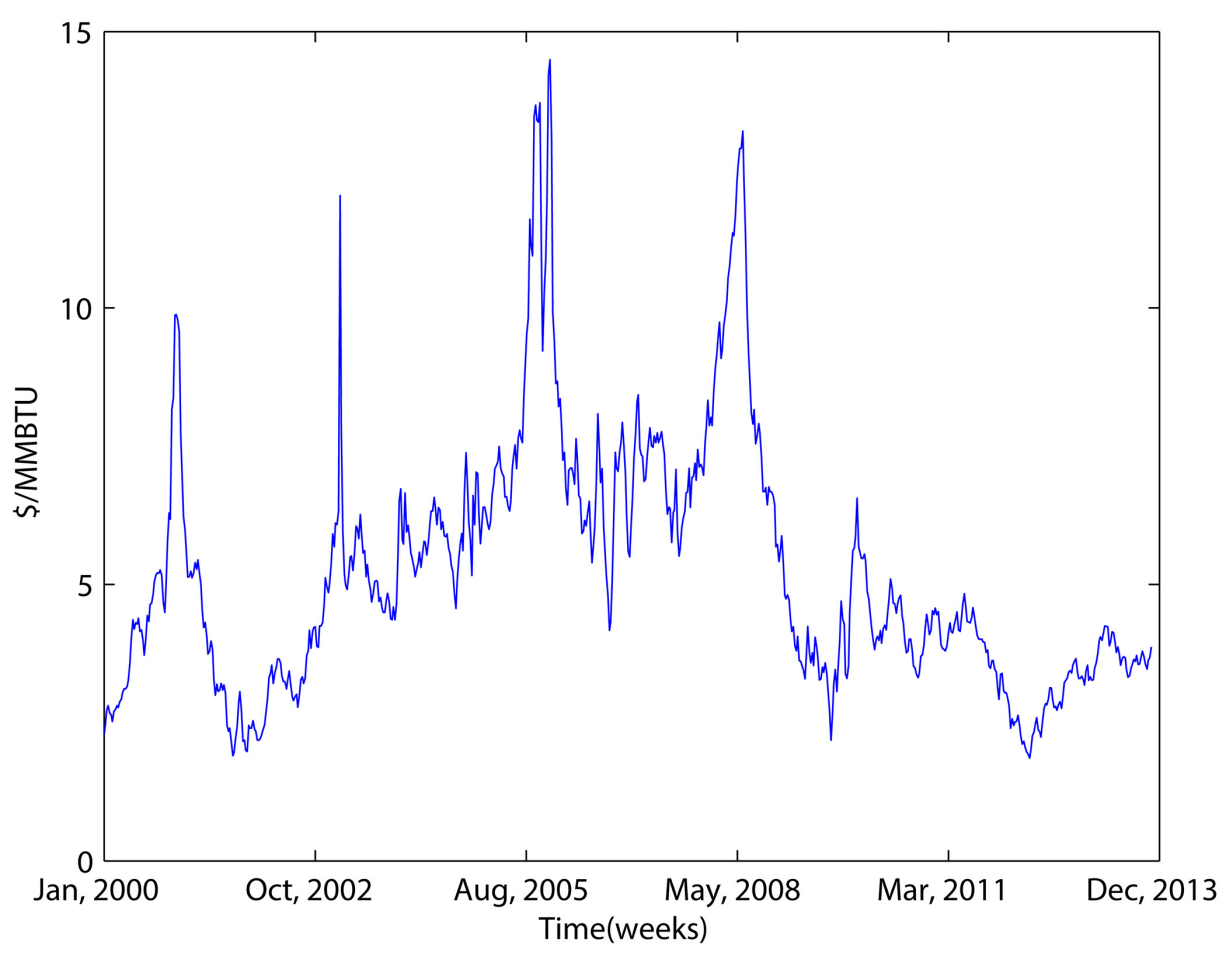
Henry Hub weekly spot prices from 2000 to 2013.

Representative international gas prices could be represented by the Henry Hub price, or the national balancing point (NBP) price. In this study we chose Henry Hub, because it is less dependent on the oil market than the NBP [51]. Henry Hub weekly spot prices were gathered for our analysis. In 2005, 44% of gas production in the United States was transferred through Henry Hub. Furthermore, Henry Hub is used as the index price for NYMEX, and for other spot and futures trading. The prices during the period extending from 2000 to November 2013 from the EIA are used in this study. Fig 6 shows the movements of the prices. The first 624 samples were used to construct forecasting models, and the rest were used to test the performance of these models; that is, out-of-sample forecasting.

### ARIMA, ANN, and ARIMA combined with wavelets and ANN combined with wavelets

In the first group of forecasting, ANN, ARIMA, wavelet decomposition combined with ANN, and wavelet decomposition combined with ARIMA were used to forecast natural gas spot prices by a multi-step ahead process. In the case where ARIMA and ANN are applied, the original data are forecasted directly. In the cases of a wavelet decomposition combination, only the approximation component was used for forecasting. The cases of wavelet decomposition at this stage do not consider the boundary problem. The optimal time lags for each case were selected by the AR term in ARIMA models. For clarity, only two models are compared in each picture. The results of the ARIMA model specification for the original series and cA3 component are in the Tables B and C in S1 File.

**Fig 7:**
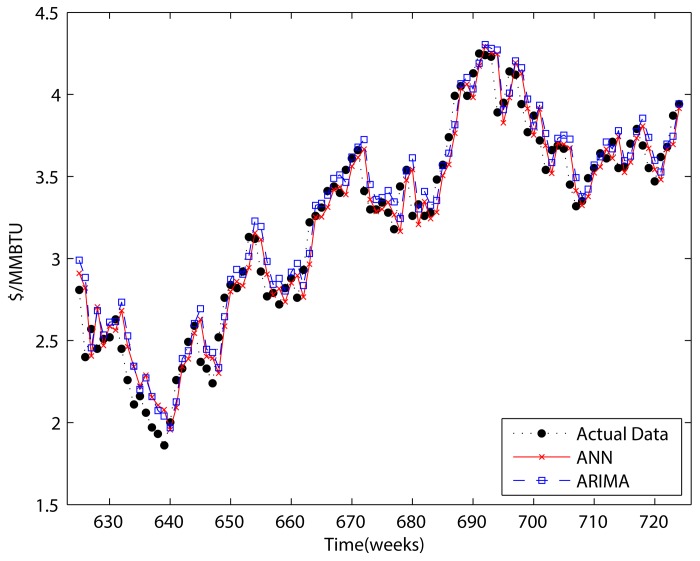
One-step forecasting results of ARIMA and ANN.

**Fig 8:**
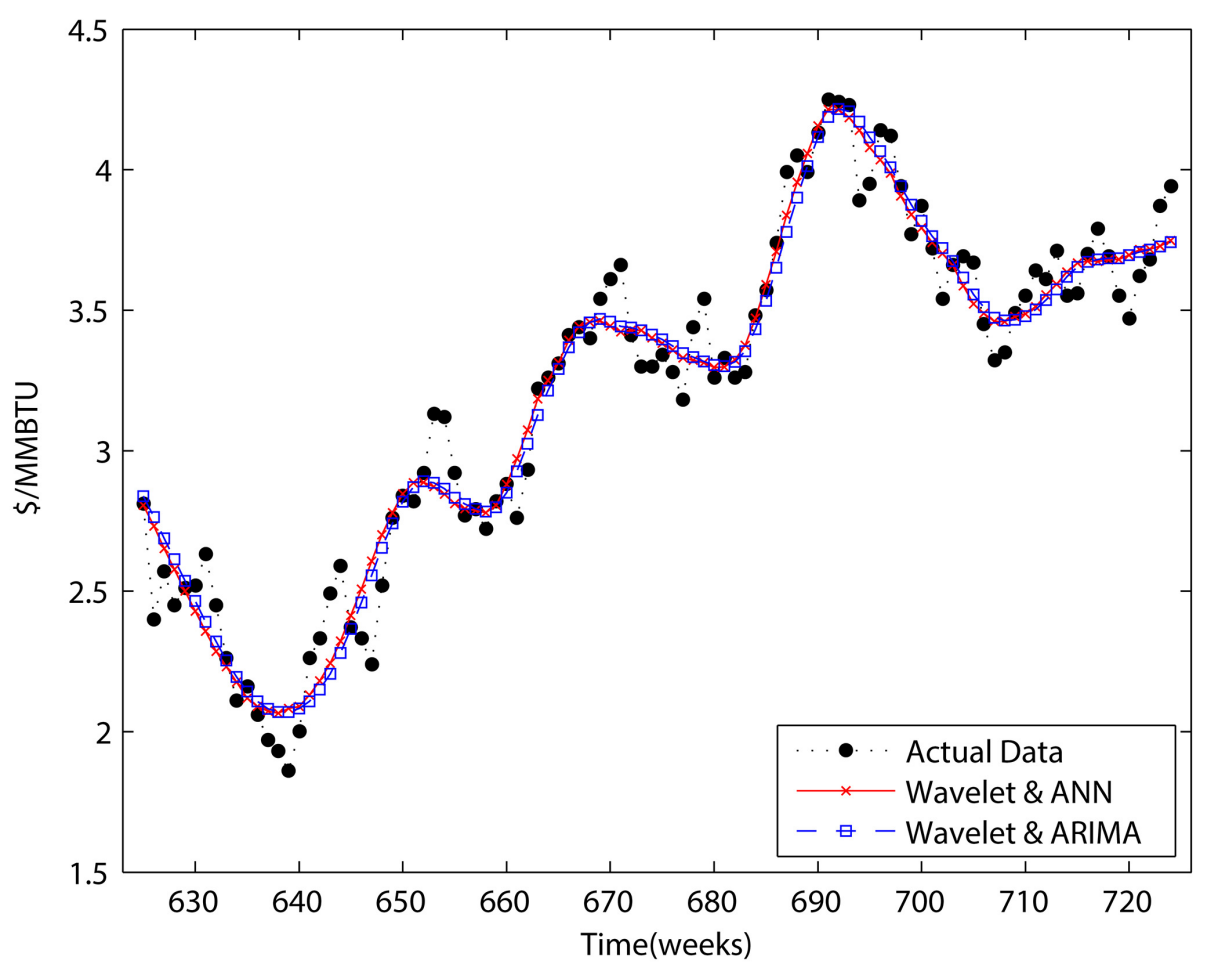
One-step forecasting results for wavelets combined with ANN and wavelets combined with ARIMA.

**Fig 9:**
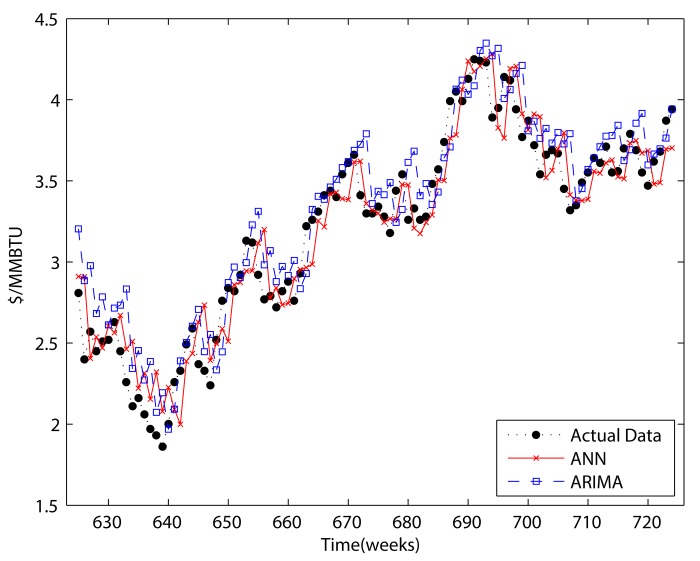
Two-step forecasting results of ARIMA and ANN.

**Fig 10:**
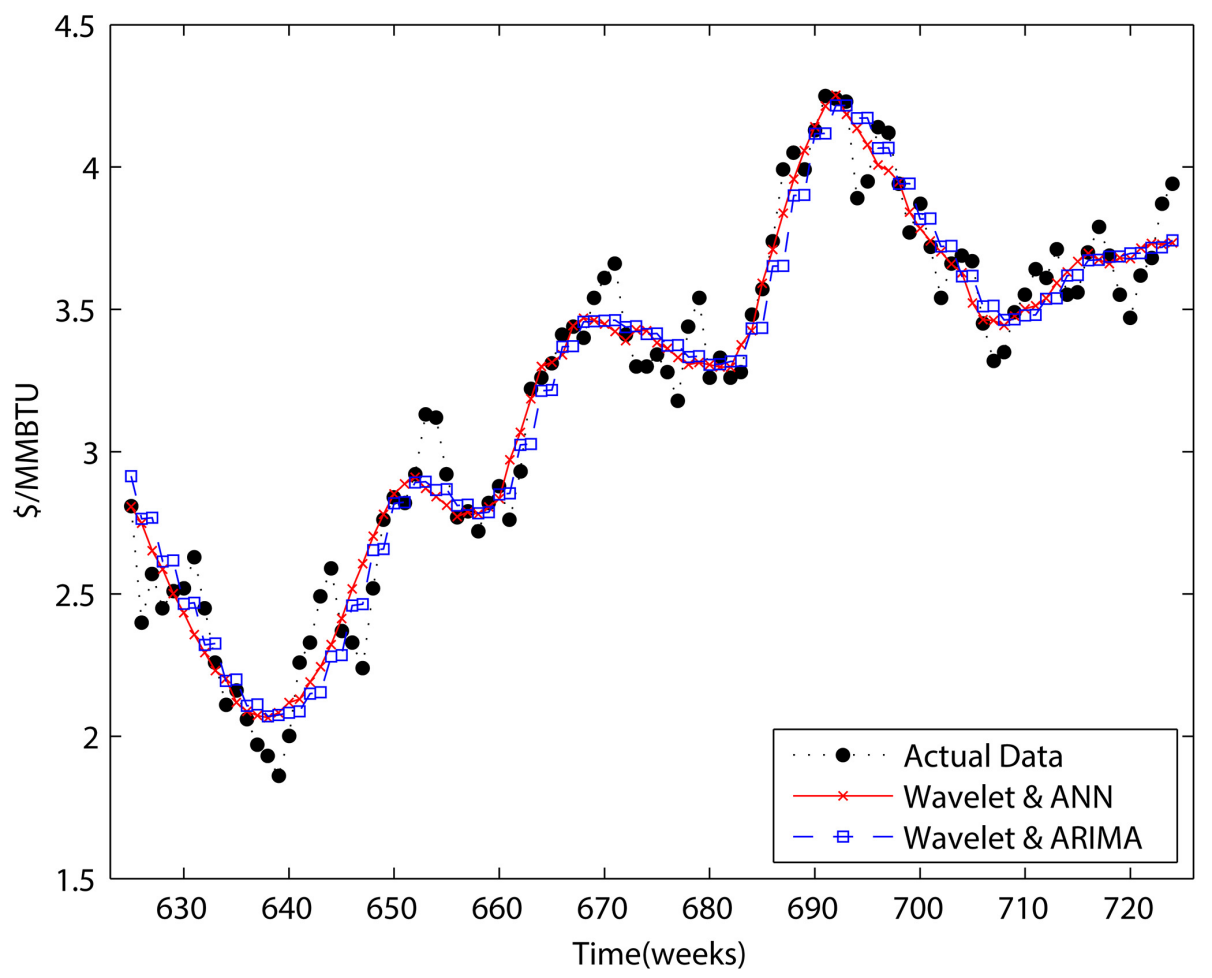
Two-step forecasting results for wavelets combined with ANN and wavelets combined with ARIMA.

Figs 7–10 show the results of ANN, ARIMA, wavelet decomposition combined with ANN, and wavelet decomposition combined with ARIMA in both one-step and two-step forecasting, respectively. As shown in the figures, ANN and ARIMA are sensitive to data fluctuations, but the cases of combinations with wavelet decomposition appear to result in a smoothed version of the original data. As shown in Tables 1 and 2, the forecasting performance decreased when the number of steps was increased. The ANN cases show a better performance than the ARIMA cases, and wavelet decomposition was able to improve the forecasting performance. This is because wavelet decomposition decomposes the original data into subseries, which creates data more suitable for forecasting. However, as we stated above regarding the boundary problem, these cases are problematic in that they use future information for forecasting purposes.

**Table 1:** Analysis of the one-step and two-step forecasting results.

	One-step			Two-step		
	RMSE*	MAE*	MAPE(%)*	RMSE	MAE	MAPE(%)
**ANN**	**0.1439**	**0.1173**	**3.9560**	**0.1884**	**0.1533**	**5.2006**
**ARIMA**	**0.1526**	**0.1192**	**4.0431**	**0.2221**	**0.1770**	**6.0253**
**Wavelet with ANN**	**0.1278**	**0.0985**	**3.3192**	**0.1289**	**0.1000**	**3.3691**
**Wavelet with ARIMA**	**0.1285**	**0.1008**	**3.3747**	**0.1366**	**0.1112**	**3.7018**

*RMSE means Root Mean Squared Errors, MAE means Mean Absolute Errors, MAPE means Mean Absolute Percentage Errors.

**Table 2:** Analysis of the three-step and four-step forecasting results.

	Three-step			Four-step		
	RMSE	MAE	MAPE(%)	RMSE	MAE	MAPE(%)
**ANN**	**0.2484**	**0.2003**	**6.8334**	**0.3070**	**0.2352**	**8.1679**
**ARIMA**	**0.2633**	**0.2009**	**6.9681**	**0.3274**	**0.2476**	**8.8125**
**Wavelet with ANN**	**0.1295**	**0.1027**	**3.4367**	**0.1312**	**0.1026**	**3.4871**
**Wavelet with ARIMA**	**0.1540**	**0.1234**	**4.0655**	**0.1753**	**0.1371**	**4.5010**

### Results of models with detail components

The second forecasting group includes detail components. These components indicate the volatility of time series, and we want to determine whether detail components are meaningful for forecasting, or whether they simply constitute noise. Tables 3 and 4 show the forecasting results with detail components. The results of the previous section indicated that approximation components should be forecast by using ANN. Therefore, the approximation components used in this section are estimated by using ANN, whereas detail components are forecast by ANN and GARCH. A comparison of the following tables enabled us to determine which model is more suitable for forecasting detail components. We did not use ARIMA to forecast the detail components because an ARIMA model generally provides good results in the absence of large random changes [44]. Furthermore, GARCH is generally applied to highly volatile series caused by unexpected random effects [17, 52]. Because detail components are high volatile signals that have heteroskedasticity, we only applied ANN and GARCH to forecast detail components, but not ARIMA. The results indicate that the detail components have a slight influence on forecasting results, in the sense that they decrease the forecasting performance when ANN is used as the forecasting method. We also applied other approaches to forecast detail components. Using Engle’s ARCH test, heteroskedasticity was found in the detail components. However, the use of GARCH led to a slight increase in the forecasting performance. Therefore, we infer two conclusions. First, GARCH is more suitable to forecast detail components. Second, even though the detail components have a little effect, its effect is very slight or negative. Therefore, it is hard to conclude that there is practical advantage when including detail components in the model. The results of ARCH test, model specification of each detail component are in the Tables D, E, F, and G in S1 File.

**Table 3:** Forecasting results including detail component forecasted by ANN (one- and two-step).

	One-step			Two-step		
	RMSE	MAE	MAPE(%)	RMSE	MAE	MAPE(%)
**cA3**	**0.1278**	**0.0985**	**3.3192**	**0.1289**	**0.1000**	**3.3691**
**cA3&cD3**	**0.1420**	**0.1152**	**3.7760**	**0.1325**	**0.1033**	**3.2996**
**cA3&cD2**	**0.1497**	**0.1159**	**3.8136**	**0.1319**	**0.1042**	**3.3884**
**cA3&cD1**	**0.1470**	**0.1121**	**3.6211**	**0.1325**	**0.1015**	**3.3151**
**cA3&cD2&cD1**	**0.1684**	**0.1298**	**4.3774**	**0.1365**	**0.1081**	**3.5751**
**cA3&cD3&cD1**	**0.1579**	**0.1240**	**4.0229**	**0.1574**	**0.1053**	**3.3750**
**cA3&cD2&cD3**	**0.1573**	**0.1249**	**4.1770**	**0.1592**	**0.1064**	**3.5378**
**cA3&cD1&cD2&cD3**	**0.1737**	**0.1374**	**4.5951**	**0.1733**	**0.1105**	**3.6216**

**Table 4:** Forecasting results including detail component forecasted by ANN (three- and four-step).

	Three-step			Four-step		
	RMSE	MAE	MAPE(%)	RMSE	MAE	MAPE(%)
**cA3**	**0.1295**	**0.1027**	**3.4367**	**0.1312**	**0.1026**	**3.4871**
**cA3&cD3**	**0.1454**	**0.1136**	**3.7173**	**0.1443**	**0.1051**	**3.7498**
**cA3&cD2**	**0.1501**	**0.1156**	**3.7602**	**0.1524**	**0.1202**	**3.9259**
**cA3&cD1**	**0.1439**	**0.1148**	**3.6986**	**0.1325**	**0.1155**	**3.4550**
**cA3&cD2&cD1**	**0.1680**	**0.1296**	**4.3389**	**0.1605**	**0.1297**	**4.3342**
**cA3&cD3&cD1**	**0.1559**	**0.1244**	**4.0248**	**0.1463**	**0.1189**	**3.8479**
**cA3&cD2&cD3**	**0.1596**	**0.1270**	**4.2227**	**0.1535**	**0.1220**	**4.0454**
**cA3&cD1&cD2&cD3**	**0.1735**	**0.1362**	**4.5503**	**0.1624**	**0.1314**	**4.3539**

### Results of adjusted forecasting methods using wavelet decomposition

As we stated in the introduction, disregarding the boundary problem will result in overestimation. Therefore, we perform adjusted two-step ahead and four-step ahead forecasting to handle the boundary problem in this section, and compare the results to those of previous sections.

We forecast the approximation by using ARIMA and ANN. Although ANN performed better than ARIMA in previous sections, ARIMA is also used to show that there is definitely an over estimation problem. In contrast, the detail components were only forecast by using GARCH because of its superior ability to forecast detail components compared to ANN.

**Fig 11:**
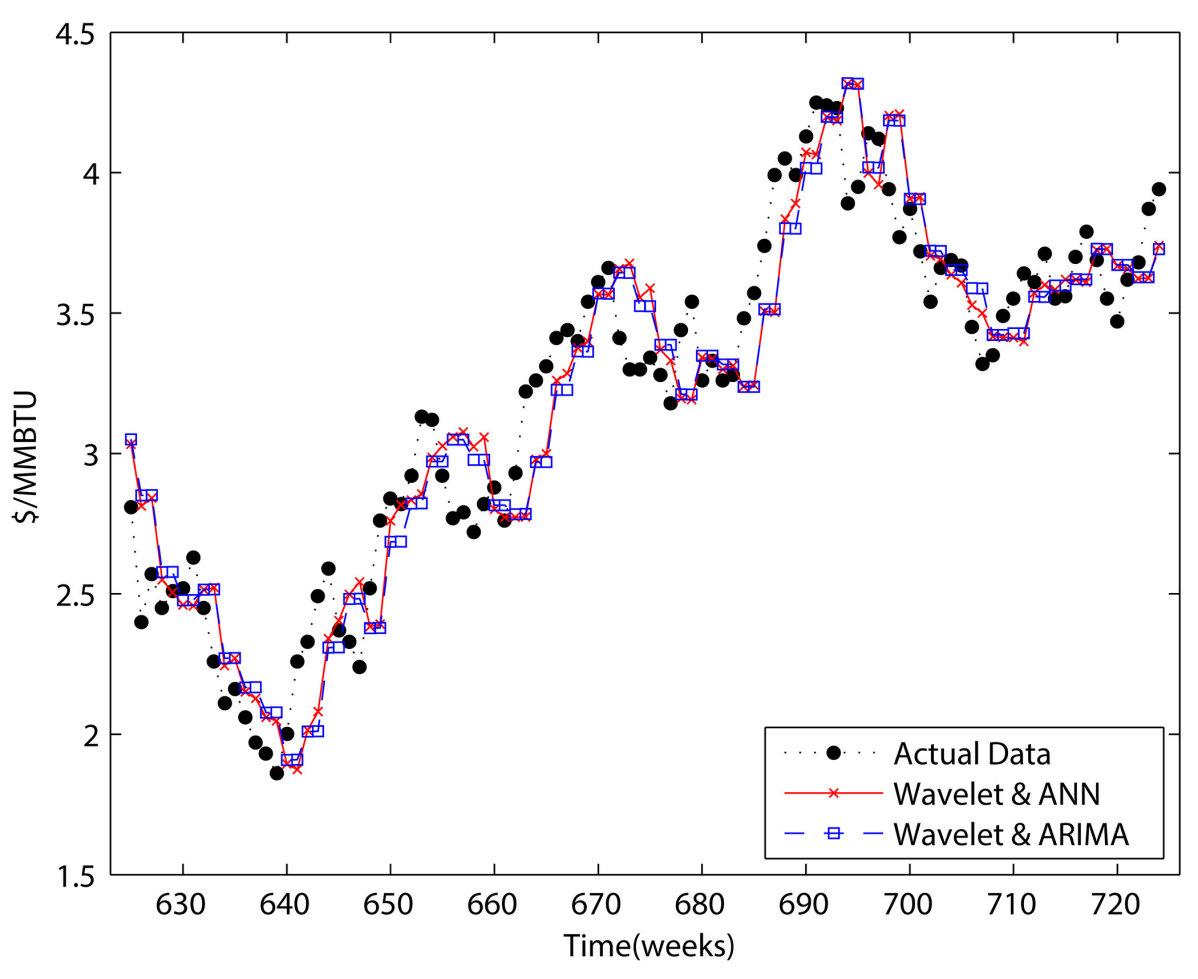
Adjusted two-step wavelet decomposition forecasting.

**Fig 12:**
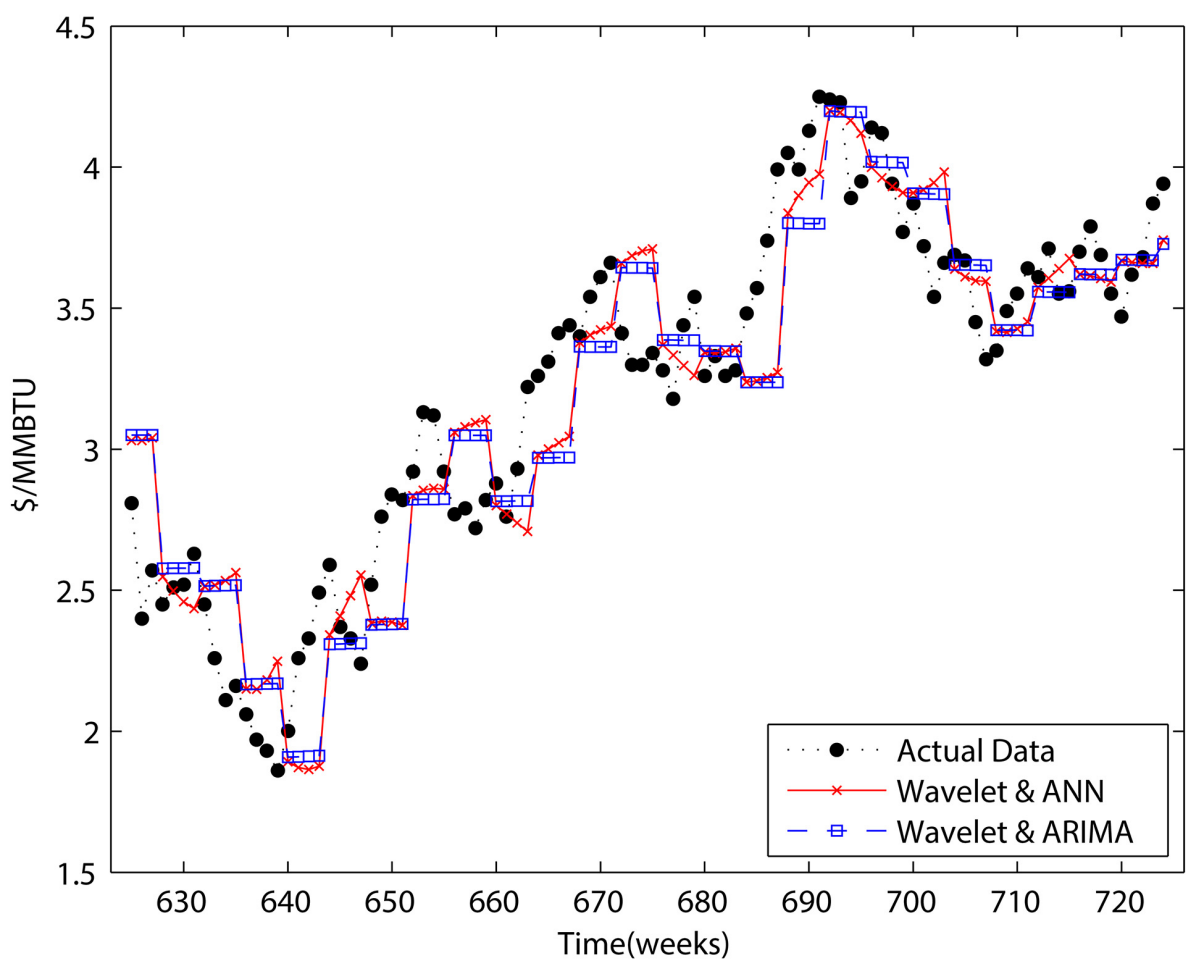
Adjusted four-step wavelet decomposition forecasting.

Figs 11 and 12 present the results for “adjusted forecasting.” Comparing them to Figs 8 and 10, we can see that the results no longer represent a smoothed version of the original data. When comparing Tables 5 and 6 to Tables 7 and 8, it becomes clear that there is a considerable overestimation of forecasting when the boundary condition is not considered. Although the forecasting performance decreases when the boundary condition is taken into account, based on comparing the results of Tables 7 and 8 to Tables 1 and 2 wavelet decomposition could improve the performance of forecasting. In respect of detail components, there is only one case which improves the forecasting performance. Furthermore, that improvement was only less than 0.1%. These results indicate that including the detail components in a forecasting model is not helpful in the perspective of forecasting power.

**Table 5:** Forecasting results including detail component forecasted by GARCH (one- and two-step).

	One-step			Two-step		
	RMSE	MAE	MAPE(%)	RMSE	MAE	MAPE(%)
**cA3**	**0.1278**	**0.0985**	**3.3191**	**0.1289**	**0.0998**	**3.3691**
**cA3&cD3**	**0.1279**	**0.0986**	**3.3071**	**0.1299**	**0.1015**	**3.3968**
**cA3&cD2**	**0.1274**	**0.0982**	**3.2821**	**0.1285**	**0.0992**	**3.3205**
**cA3&cD1**	**0.1271**	**0.0980**	**3.2813**	**0.1282**	**0.0990**	**3.3200**
**cA3&cD2&cD1**	**0.1271**	**0.0980**	**3.2714**	**0.1280**	**0.0990**	**3.3111**
**cA3&cD3&cD1**	**0.1274**	**0.0983**	**3.2949**	**0.1291**	**0.1008**	**3.3704**
**cA3&cD2&cD3**	**0.1279**	**0.0985**	**3.2930**	**0.1295**	**0.1009**	**3.3670**
**cA3&cD1&cD2&cD3**	**0.1277**	**0.0986**	**3.2929**	**0.1291**	**0.1005**	**3.3483**

**Table 6:** Forecasting results including detail component forecasted by GARCH (three- and four-step)

	Three-step			Four-step		
	RMSE	MAE	MAPE(%)	RMSE	MAE	MAPE(%)
**cA3**	**0.1295**	**0.1027**	**3.4367**	**0.1312**	**0.1026**	**3.4871**
**cA3&cD3**	**0.1297**	**0.1027**	**3.4263**	**0.1308**	**0.1022**	**3.4311**
**cA3&cD2**	**0.1290**	**0.1021**	**3.3931**	**0.1302**	**0.1017**	**3.4076**
**cA3&cD1**	**0.1290**	**0.1020**	**3.3973**	**0.1302**	**0.1016**	**3.4124**
**cA3&cD2&cD1**	**0.1289**	**0.1017**	**3.3751**	**0.1294**	**0.1013**	**3.3888**
**cA3&cD3&cD1**	**0.1294**	**0.1022**	**3.4043**	**0.1299**	**0.1014**	**3.4011**
**cA3&cD2&cD3**	**0.1295**	**0.1022**	**3.3992**	**0.1300**	**0.1015**	**3.3960**
**cA3&cD1&cD2&cD3**	**0.1295**	**0.1021**	**3.3910**	**0.1294**	**0.1011**	**3.3776**

**Table 7:** Results for the adjusted decomposition without detail components.

	Two-step			Four-step		
	RMSE	MAE	MAPE(%)	RMSE	MAE	MAPE(%)
**Wavelet with ANN**	**0.2090**	**0.0066**	**5.6437**	**0.2628**	**0.0185**	**7.1775**
**Wavelet with ARIMA**	**0.2124**	**0.0103**	**5.8580**	**0.2621**	**0.0256**	**6.9971**

**Table 8:** Forecasting results including detail components by adjusted decomposition and GARCH.

	****Two-step****			****Four-step****		
	****RMSE****	****MAE****	****MAPE(%)****	****RMSE****	****MAE****	****MAPE(%)****
**cA3**	**0.2090**	**0.0066**	**5.6437**	**0.2621**	**0.0256**	**6.9971**
**cA3&cD3**	**0.2100**	**0.0080**	**5.6347**	**0.2636**	**0.0229**	**7.0477**
**cA3&cD2**	**0.2111**	**0.0092**	**5.6537**	**0.2682**	**0.0260**	**7.1403**
**cA3&cD1**	**0.2117**	**0.0084**	**5.7239**	**0.2606**	**0.0225**	**6.9136**
**cA3&cD2&cD1**	**0.2145**	**0.0110**	**5.7535**	**0.2684**	**0.0280**	**7.1372**
**cA3&cD3&cD1**	**0.2130**	**0.0098**	**5.7399**	**0.2644**	**0.0262**	**7.7020**
**cA3&cD2&cD3**	**0.2127**	**0.0105**	**5.6781**	**0.2718**	**0.0277**	**7.1974**
**cA3&cD1&cD2&cD3**	**0.2164**	**0.0123**	**5.7765**	**0.2740**	**0.0298**	**7.2711**

## Conclusion

Based on a combination of wavelet decomposition with the ANN, ARIMA, and GARCH models, we have suggested an up-to-date forecasting model for natural gas prices. Our proposed approach can handle the boundary problem, such that it facilitates the extraction of the appropriate forecasting results. In addition, our tests showed that the inclusion of detail components improved the forecasting performance, even though the improvement was slight, and this indicated that detail components do not merely constitute noise.

A comparison of the results in Tables 1 and 5 with those in Tables 7 and 8 showed that there is definitely an over estimation problem when the boundary problem is not considered. In terms of forecasting performance, wavelet decomposition was not found to improve the performance of two-step ANN forecasting; however, the results of ARIMA forecasting were improved slightly. Many prior studies have shown that the best performance is obtained by using wavelet decomposition combined with ANN. However, in our analysis, using ANN alone was shown to be the best method for two-step forecasting, whereas wavelet decomposition combined with ARIMA was the best case for four-step forecasting. Therefore, the classical ARIMA forecasting methodology should be considered as an alternative choice when applying wavelet decomposition.

The results we obtained for the adjusted forecasting enabled us to reach the conclusion that GARCH is more suitable than ANN to forecast the detail component but the inclusion of the detail component in a forecasting model do not offer a clear advantage against the model in which only the approximation component. Table 8 which contains the forecasting results considering the boundary condition supports this conclusion. Therefore, when forecasting natural gas prices, usage of de-noised time series which means the approximation components of time series can be an appropriate alternative.

Our proposed approach for discrete wavelet decomposition is simple, and is capable of simplifying the data processing procedure. However, the properties of discrete wavelet decomposition, or time-variant transformation prevented us from carrying out odd-number step-ahead forecasting. In addition, the mirror-effect problem, a problem associated with many wavelets other than the Harr wavelet, also remains. We will address these issues in future work.

## Supporting Information

S1 File Table A- Optimal hidden node and lags for ANN modeling; Table B- Results of ARIMA for the original series; Table C- Results of ARIMA for cA3; Table D- Results of LM-ARCH test; Table E- Results of GARCH(3,3) for cD1; Table F- Results of GARCH(1,2) for cD2; Table G- Results of GARCH(1,2) for cD3. (DOC)
