# Supplementary material for: Forecasting Natural Gas Prices Using Wavelets, Time Series, and Artificial Neural Networks
Source: PLoS One. 2015 Nov 5;10(11):e0142064. doi: 10.1371/journal.pone.0142064 (PMC4635006; doi:10.1371/journal.pone.0142064)
Supplement: S1 File — (DOC) [file pone.0142064.s001.doc]

**Supporting Information.**

In this section, we show all optimal number of hidden nodes for ANN models and results of ARIMA and GARCH model specification.

Table A. Optimal hidden node and lags for ANN modeling

| **Series** | **Original** | **cA3** | **cD3** | **cD2** | **cD1** |
| --- | --- | --- | --- | --- | --- |
| Number of optimal hidden node | 3 | 4 | 5 | 4 | 3 |
| Optimal time-lags | 3 | 3 | 2 | 2 | 3 |

Table B1. Results of ARIMA for the original series

|  | **AR(1)** | **AR(2)** | **AR(3)** | **MA(1)** | **MA(2)** | **MA(3)** |
| --- | --- | --- | --- | --- | --- | --- |
| Coefficient | 2.3814 | -2.0121 | 0.6299 | -1.3005 | 0.5093 | 0.0099 |
| Standard error | 0.3775 | 0.6161 | 0.2444 | 0.3762 | 0.1940 | 0.0857 |
| p-value | 2.8152e-10 | 1.0919e-03 | 9.9418e-03 | 5.4599e-04 | 8.6555e-03 | 9.0809-e01 |

Table C2. Results of ARIMA for cA3

|  | **AR(1)** | **AR(2)** | **AR(3)** | **MA(1)** |
| --- | --- | --- | --- | --- |
| Coefficient | 2.9099 | -2.8836 | 0.9737 | 1.0000 |
| Standard error | 0.0083 | 0.0164 | 0.0083 | 0.0036 |
| p-value | 0.0000 | 0.0000 | 0.0000 | 0.0000 |

Table D. Results of LM-ARCH test

|  | **Test-statistics** | **p-value** |
| --- | --- | --- |
| cD1 | 70.8551 | 2.2153e-10 |
| cD2 | 199.785 | 0.0000 |
| cD3 | 240.9362 | 0.0000 |

Table E. Results of GARCH(3,3) for cD1

|  | Constant | $\alpha_{1}$ | $\alpha_{2}$ | $\alpha_{3}$ | $\delta_{1}$ | $\delta_{2}$ | $\delta_{3}$ |
| --- | --- | --- | --- | --- | --- | --- | --- |
| Coefficient | 0.0001 | 0.5607 | -0.6517 | 0.3430 | 1.4801 | -0.9911 | 0.2897 |
| Standard error | 5.97e-05 | 0.0876 | 0.1399 | 0.0896 | 0.1636 | 0.2470 | 0.1207 |
| Z-statistics | 2.7560 | 6.3976 | -4.6583 | 3.8276 | 9.0490 | -4.0124 | 2.3995 |
| p-value | 0.0059 | 0.0000 | 0.0000 | 0.0001 | 0.0000 | 0.000 | 0.0164 |

Table F. Results of GARCH(1,2) for cD2

|  | Constant | $\alpha_{1}$ | $\delta_{1}$ | $\delta_{2}$ |
| --- | --- | --- | --- | --- |
| Coefficient | 0.0003 | 0.7661 | -0.5556 | 0.7955 |
| Standard error | 0.0001 | 0.0481 | 0.0653 | 0.0369 |
| Z-statistics | 2.6556 | 15.9377 | -8.5116 | 21.5647 |
| p-value | 0.0079 | 0.0000 | 0.0000 | 0.000 |

Table G. Results of GARCH(1,2) for cD3

|  | Constant | $\alpha_{1}$ | $\delta_{1}$ | $\delta_{2}$ |
| --- | --- | --- | --- | --- |
| Coefficient | 1.53e-05 | 0.9442 | -0.7965 | 0.8577 |
| Standard error | 1.19e-05 | 0.0317 | 0.0491 | 0.0336 |
| Z-statistics | 1.2919 | 29.8013 | -16.2139 | 25.5080 |
| p-value | 0.1964 | 0.0000 | 0.0000 | 0.000 |
